# Primary Fallopian Tube Carcinoma: An Extremely Rare Gynecological Cancer Misdiagnosed Intraoperatively as Benign Ovarian Neoplasm: A Case Report

**DOI:** 10.3390/clinpract12030030

**Published:** 2022-04-22

**Authors:** Efthymia Thanasa, Dimitra Stamouli, Ektoras-Evangelos Gerokostas, Konstantina Balafa, Nikoleta Koutalia, Ioannis Thanasas

**Affiliations:** 1 Department of Health Sciences, Medical School, Aristotle University of Thessaloniki, 54124 Thessaloniki, Greece; effiethan@gmail.com; 2 Department of Obstetrics and Gynecology, General Hospital in Trikala, Efkli 33, 42100 Trikala, Greece; dimistam37@gmail.com (D.S.); hectorger21@gmail.com (E.-E.G.); balafa_konstantina@hotmail.com (K.B.); kout_95@yahoo.gr (N.K.)

**Keywords:** fallopian tube, primary cancer, surgery, examination of the frozen section, chemotherapy

## Abstract

Primary fallopian tube carcinoma is very rare. Diagnosis is challenging. The description of our case concerns an asymptomatic 71-year-old patient who came for a routine gynecological examination. Imaging of the pelvis revealed the presence of a two-chambered cystic formation in the anatomical position of the right ovary. It was decided to investigate the disease by laparotomy. Examination of the frozen section from the site of the cystic lesion was negative for malignancy. An abdominal total hysterectomy was performed with bilateral salpingo-oophorectomy. Serous carcinoma of the fallopian tube was diagnosed postoperatively by histological examination of the surgical preparation. Immediately after surgery, the patient’s health was good.The patient was referred to an oncology center and was monitored. Chemotherapy based on platinum and taxane was recommended. Six months after the operation the patient is in good health. The possibility of a second surgery to treat fallopian tube cancer with pelvic lymph node dissectionis under discussion and is expected to be decided by oncologists and gynecologists-oncologists. In this article, after describing the case report, a brief review of this rare entity disease’s diagnostic and therapeutic approach is attempted.

## 1. Introduction

Primary fallopian tube carcinoma is an extremely rare gynecological cancer. It usually affects patients of postmenopausal age, with a peak incidence between at the ages of 60 and 64 years [1,2]. It was first described as a separate nosological entity by Rokitansky in 1847. Primary fallopian tube carcinoma is estimatedto concern 0.14–0.18% of all genital cancers [3], although many today argue that the incidence of fallopian tube cancer is significantly underestimated [4]. The etiology has not been fully clarified. Hormonal, reproductive, and potential genetic factors believed to increase the risk of ovarian epithelial cancer have also been included in the etiologic mechanism of primary fallopian tube cancer [5]. The prognosis depends on the stage of the disease at diagnosis, the histological type of the cancer, and the degree of success of the cytoreductive surgery. At an advanced stage of the disease, the five-year survival is low (34%) [6,7].

This case study highlights the significant preoperative and intraoperative difficulties in the diagnostic approach of tubal cancer patients, including the frozen section examination. Careless examination of the frozen section and misdiagnosis of primary fallopian tube carcinoma as a benign ovarian/adnexal mass significantly affects the early diagnosis andthe most appropriate surgical approachto the disease, the correct application of which can lead to the best prognostic outcome for these patients.

## 2. Case Presentation

**Figure 1:**
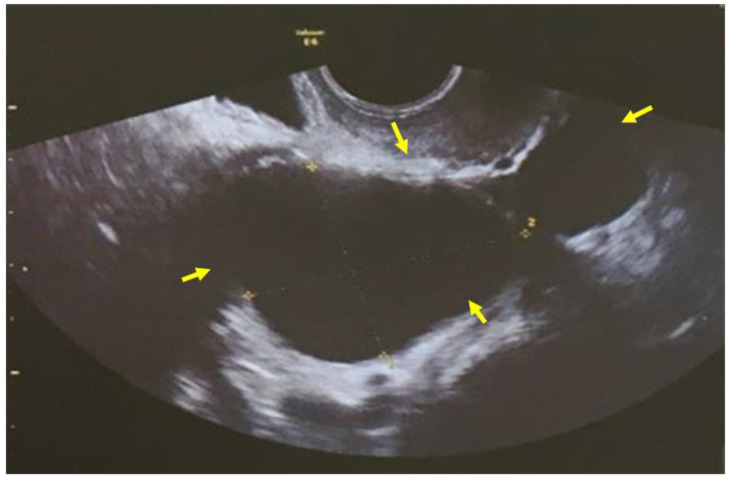
Transvaginal ultrasound imaging: the allantoid form cystic mass at the anatomical position of the right adnexa (yellow arrows) corresponding to primary fallopian tube carcinoma was misdiagnosed as a two-chambered cystic lesion of the ovary (our case).

**Figure 2:**
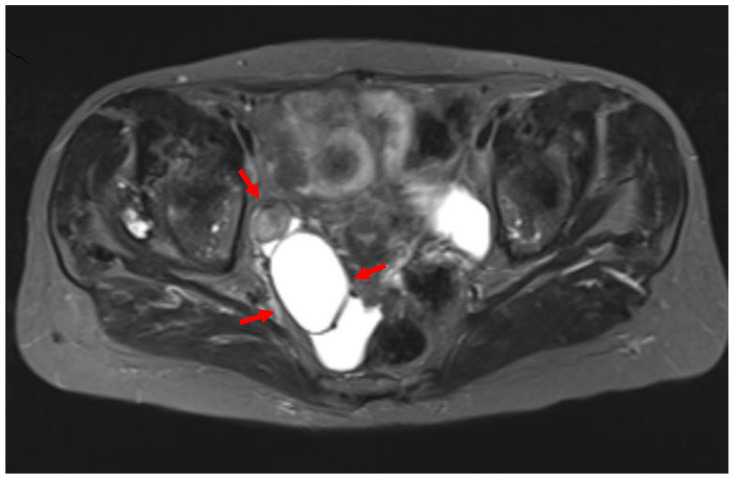
Magnetic resonance imaging of primary carcinoma of the fallopian tube (red arrows), which both preoperatively and intraoperatively was misdiagnosed as a benign ovarian tumor (our case).

A 71-year-old patient came for a routinegynecological examination. The patient was asymptomatic. Her personal medical history reported no health problems, other than high blood pressure. She and her family emphasized the genetic medical history of gynecological cancer: her mother and sister died at the age of 82 and 74, respectively, diagnosed with endometrial cancer endometrioid type. On transvaginal ultrasound, the uterus was of normal size. The presence of a two-chambered cystic formation at the anatomical position of the right ovary with a maximum diameter of about 6 cm was confirmed (Figure 1). Magnetic resonance imaging confirmed the ultrasound findings. Two-chamber cystic formation of increased intensity in the T2 sequences was identified in the area of the right adnexa. After intravenous administration of paramagnetic shading agent, no image of enrichment from the cystic lesion was identified (Figure 2). Malignancy markers (CEA, CA125, CA15-3, CA19-9) were negative.

**Figure 3:**
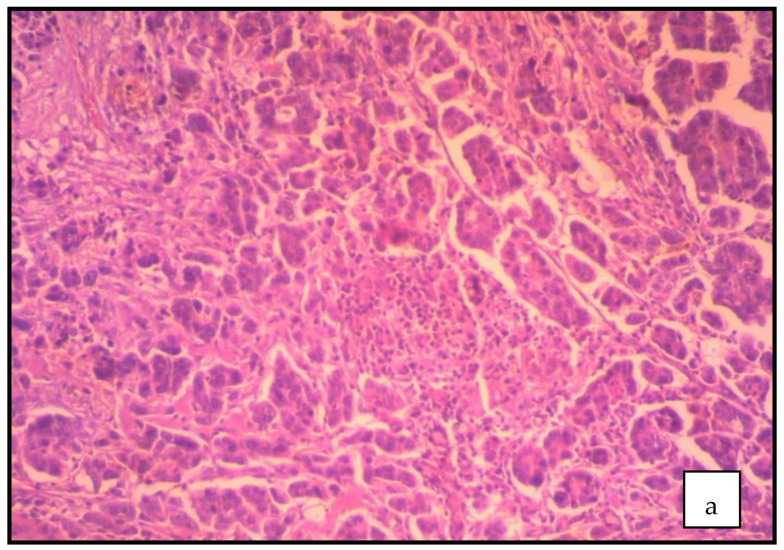

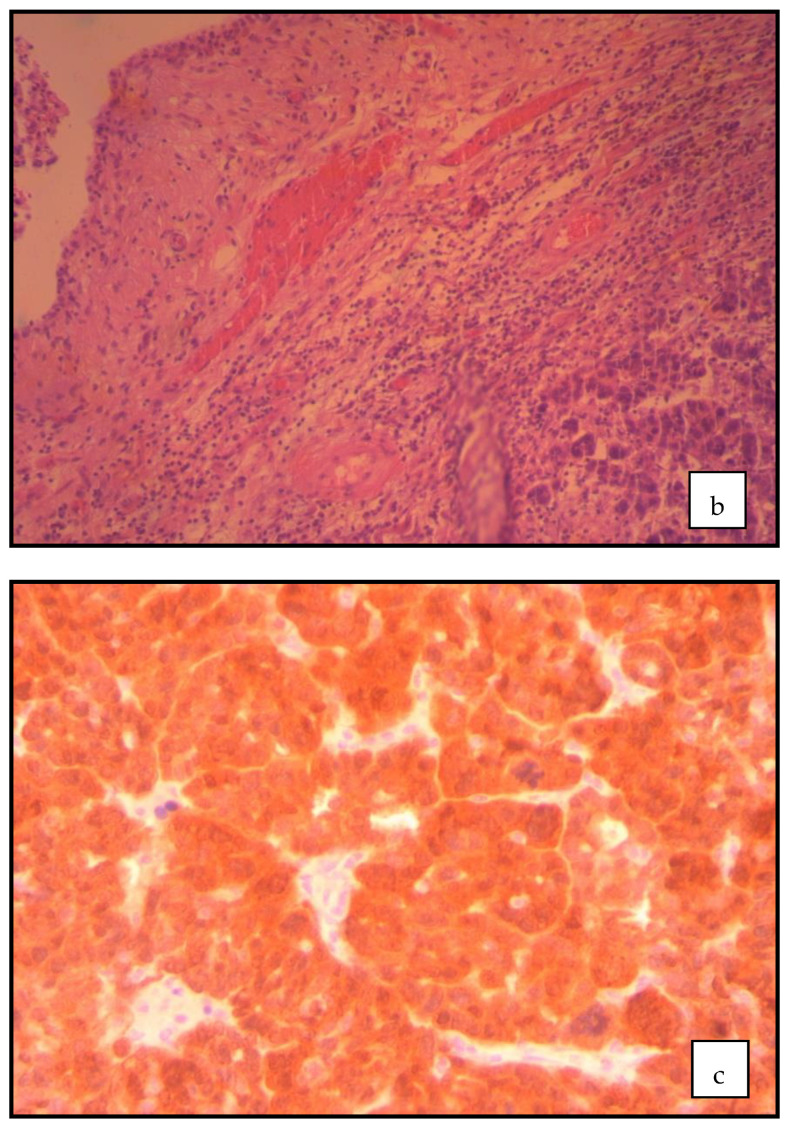
(**a**–**c**) Pathological examination of the surgical preparation (our case): (**a**) primary malignant neoplasm of high malignancy with sufficient polymorphism, atypical mitoses and necrosis; (**b**) the tumor develops in the fallopian tube epithelium followed by the epithelium of a fringe at the bell end of the fallopian tube; (**c**) immunohistochemistry.

The absence of typical imaging findings characterizing benign ovarian neoplasms and the patient’s insistence on surgery based on a positive family history of gynecological cancer led to the decision toinvestigate the pelvic mass surgically. The ovarian mass was removed intact and submitted to pathology for frozen section examination. The result of the intraoperative histopathological examination was negative for malignancy. An abdominal total hysterectomy was performed with bilateral salpingo-oophorectomy. The cytological examination of the peritoneal lavage was negative for malignancy. Postoperative, the histological examination of the surgical preparation diagnosed serous carcinoma of the fallopian tube. Macroscopic examination showed a descriptive formation with a maximum diameter of 2.5 cm in the right fallopian tube. Respectively, a seromucous cystadenoma of the ovary with a maximum diameter of 7 cm with repressed ovarian tissue in the periphery was found. On microscopic examination, a serous carcinoma of high-grade malignancy with sufficient polymorphism, atypical mitoses, and necrosis was described (Figure 3a). The tumor develops in the fallopian tube epithelium followed by the epithelium of a fringe at the bell end of the fallopian tube (Figure 3b). The immunohistochemical study (Figure 3c) showed: WT1+++, ER+++, CK7+++, p16+++, and ki~45%.

Immediately after surgery, the patient’s health was good. The patient was discharged five days after the surgery.She was then referred to an oncology center and placed under medical supervision.Platinum and taxane-based chemotherapy was recommended. Our patient with a positive family history of endometrial cancer did not have genetic counseling. It was strongly suggested that genetic testing be performed, which should mainly involve the BRCA1 and BRCA2 genes. Six months after the operation the patient is in good health. The possibility of a second surgery to treat fallopian tube cancer with pelvic lymph node dissection is under discussion and is expected to be decided by oncologists and gynecologists-oncologists.

## 3. Discussion

Preoperative diagnosis of primary fallopian tube cancer is infrequent. The clinical signs and symptoms are unclear. In many cases, similar to our patient, the disease isasymptomaticand the diagnosis is made postoperatively in women who undergo surgery for adnexal mass [8]. The most common symptoms (Latzko triad) are abdominal pain and abnormalvaginal bleeding or discharge accompanied by the presence of a pelvic/adnexal mass [9,10]. A unique case of tubal malignancy first detected by endometrial curettage has been described [11]. Although postmenopausal bleeding with a negative diagnostic endometrial curettage, unexplained or persistent vaginal discharge, and Pap smear showing abnormal cells or glands that alternate with a negative smear should raise strong suspicions of the presence of primary fallopian tube cancer [12], the presence of ascites is an indication of advanced disease [13].

Preoperative diagnosis using imaging is not pathognomonic, and ultrasound is non-specific in the diagnosis of tubal cancer. Ultrasound-imaging cyst-shaped lesions of allantoid form with papillary adhesions and neovascularization with low resistance indices should be differentiated from hydrosalpinx, tube-ovarian abscess, or ovarian cancer. Inour case, primary fallopian tube cancer was misdiagnosed as a two-chambered cystic formation of the ovary with both transvaginal ultrasound and magnetic resonance imaging. Three-dimensional Doppler ultrasound could improve diagnostic accuracy and at the same time allow a better assessment of the fallopian tube wall and the chaotic vascular architecture characterized by fallopian tube carcinoma [14,15]. Computed tomography findings, such as stable papillary projections within the mass, support the diagnosis [16]. The findings from magnetic resonance imaging are similar, and this may help differentiate primary fallopian tube from ovarian epithelial cancer [17].

Histological examination of the surgical preparation sets the definitive diagnosis. Due to the rarity of the tumor, intraoperative diagnosis is difficult. Therefore, it is estimated that in about 27% of cases, the intraoperative diagnosis may be incorrect [18]. In our case, intraoperative histopathological examination was negative for malignancy and primary fallopian tube cancer diagnosis was confirmed postoperatively. This is common in the literature because adnexal tumors are often inaccurately diagnosed by the frozen section examination [19]. Histopathologically, serous papillary carcinoma of the fallopian tube is the most common histological type, followed by endometrioid carcinoma [20]. Necessary pathological criteria for correct diagnosis, as initially defined by Hu and his associatesin 1950 [21] and later revised by Sedlis in 1961 [22] and 1978 [23], include: (1) the primary tumor arises from the endosalpinx; (2) the histologic pattern reproduces the epithelium of the tubal mucosa; (3) histologically proven transition from benign to malignant fallopian tube epithelium; (4) the ovaries and endometrium are standard or contain fewer tumors than in the tube [13,24].

Significant preoperative and intraoperative difficulties in diagnosing primary fallopian tube cancer make it imperative today to seek diagnostic markers and protocols that are expected to be reliable diagnostic solutions during the preoperative course of these patients. Additional diagnostic tools, more accurate than an abdominal CT scan and less invasive than a diagnostic laparoscopy, may be future solutions in the diagnostic approach of tubal carcinoma [25]. The use of FDG-PET/CT (fluorodeoxyglucose-positron emission tomography/computed tomography) has been tested in the search and evaluation of incomplete tumor removal; however, the data to date cannot support the examination as a routine diagnostic examination in daily clinical practice [25]. A recent study suggests that complex examination, including cytological examination of abnormal vaginal discharge, tumor markers, computed tomography, magnetic resonance imaging, PET-CT, and laparoscopy, may significantly help in the diagnosis and to avoid inadequate surgery [26].

The multidisciplinary therapeutic approach of the disease with the collaboration of many medical specialties, such as gynecology, oncology, internal medicine, pathology, genetic counseling, and molecular biology is considered necessary. The treatment approach for primary fallopian tube cancer is the same as for epithelial ovarian cancer and peritoneal cancer and depends on the stage of the disease [27]. In early stage patients the prognosis is good and the disease can be treated [28]. Surgical treatment of patients with advanced stage disease (III or IV) should be performed by a specialized team of gynecological oncologists, in order to achieve optimal cytoreductive surgery (maximum diameter of residual disease < 1 cm) [29]. In intraoperatively confirmed cases, a total abdominal hysterectomy with bilateral salpingo-oophorectomy and infracolic omentectomy, appendectomy, peritoneal washing, and peritoneal biopsy seems to be the appropriate treatment [30]. Ιn our patient, the surgical treatment was limited to a total abdominal hysterectomy with bilateral salpingo-oophorectomyand simultaneous removal of the cystic lesion from the right adnexa area, as the disease was not diagnosed during surgery.

Complete surgical resection of the disease, including pelvic lymphadenectomy followed by adequate cycles of postoperative chemotherapy based on the combination of platinum and taxane, is an important strategy to improve the prognosis of patients [31]. The postoperative radiotherapy that has been used from time to time did not have the expected results [10]. Conventional chemotherapy is recommended for the intravenous administration of drugs once every 3 weeks for 6 cycles.In addition, intraperitoneal administrationof chemotherapeutic drugs seems to improve the prognosis, especially in patients who have undergone successful cytoreductive surgery [32]. In our patient, performing only a total abdominal hysterectomy requires reoperation in order to remove the pelvic lymph nodes. This view may be at odds with recent studies, showing that the value of lymphadenectomy in the overall five-year survival of tubal cancer is questionable and needs to be reconsidered [28,33].

Additionally, the use of modern complementary surgical techniques (ablation, ultrasonic aspiration) that can be integrated during primary surgery does not appear to improve the rate of optimal cytoreduction [34].In recent years, the benefits of the use of hyperthermic intraperitoneal chemotherapy in cytoreductive surgery have been shown to be significant in the treatment of primary fallopian tube carcinoma.The addition of hyperthermic intraperitoneal chemotherapy to surgery is estimated to increase overall patient survival without recurrence and with fewer side effects [35]. Moreover, the use of polymerase inhibitors may be effective in patients with mutations in the BRCA1 and BRCA2 genes in which they may prolong survival [36].

Testing for the presence of a pathogenic variant in the BRCA genes is important. Genetic counseling and genetic testing should be recommended nowadays for all patients diagnosed with ovarian cancer, fallopian tube cancer, or peritoneal cancer, regardless of age or family history. A recent study showed that the incidence of germline BRCA1 and BRCA2 mutations in women with high-grade serous carcinoma of the fallopian tubes and ovaries and primary peritoneal carcinoma is twice as high as in patients with negative intraepithelial carcinoma. The same authors in the same study showed that a positive family history of “BARCA-related” cancers were seen at a higher proportion in the mutation positive women [37]. It is also estimated that of patients with non-mucosal epithelial cancers of the fallopian tubes and ovaries, about 15% carry an inherited mutation in the BRCA1 and BRCA2 cancer susceptibility genes. Therefore, non-referral of these patients for genetic testing results in missed opportunities to treat the disease with more targeted therapies and failure to prevent future cancers in the patient and at-risk relatives [38]. To date, no genetic testing has been performed on our patient. Because the cost of the exams is high, and because these exams are not offered by the national health system in Greece, the results of the exams are expected.

## 4. Conclusions

Primary fallopian tube cancer is a challenge in surgical practice. Due to the rarity of the tumor and the increasing incidence in recent years, careful intraoperative histopathological examination is of great importance to avoid misdiagnosis, avoiding a new surgery. The difficult diagnosis with the existing preoperative and intraoperative diagnostic methods makes it necessarynowadays to identify new diagnostic indicatorsthat will contribute to the timely and correct diagnostic and therapeuticapproach of the disease and will significantly improve the prognosis. However, their establishment requires further experimental and clinical studies involving a larger number of cases in order to gather useful information about this rare disease.

## Data Availability

The datasets used and/or analysed during the current study are available from the corresponding author upon reasonable request.
